# EHMTI-0272. Microvascular decompression of the trigeminal nerve in the treatment of medically intractable SUNCT and SUNA

**DOI:** 10.1186/1129-2377-15-S1-C34

**Published:** 2014-09-18

**Authors:** G Lambru, S Miller, N Kitchen, L Zrinzo, M Matharu

**Affiliations:** 1 Headache Group, UCL Institute of Neurology, London, UK; 2 Headache Group, UCL Institute of Neurology and The National Hospital for Neurology and Neurosurgery, London, UK; 3 Neurosurgery, The National Hospital for Neurology and Neurosurgery, London, UK

## Introduction

Microvascular decompression (MVD) of the trigeminal nerve has been so far reported to be effective in 12/19 SUNCT and SUNA patients (63 %) with neurovascular compression.

## Aims

We report the outcome of MVD, using Jannetta procedure, in nine cases of medically intractable chronic SUNCT and SUNA patients.

## Methods

Nine patients with chronic SUNCT and SUNA with a vascular loop indenting or distorting the trigeminal nerve ipsilaterally to the side of the pain demonstrable on MRI scan were offered MVD of the trigeminal nerve. All the patients failed to respond or tolerate adequate dosages of: lamotrigine, topiramate, oxcarbazepine, carbamazepine, duloxetine, pregabalin or gabapentin and greater occipital nerve blocks. Intravenous lidocaine improved the pain only during infusion in some of them.

## Results

Seven SUNCT and two SUNA patients underwent MVD of the trigeminal nerve. They all had multiple daily severe headache attacks at baseline. At a median follow-up of 15 months (range: 8-30 months) after the operation, four patients became and remained pain free, one patient each had 90%, 70% and 50% improvement, respectively. The patient who improved by 50% was rendered pain free on preventive treatments. Two patients became pain free for 12 months, before pain returned. One patient suffered a CSF leak after the procedure.

## Conclusions

MVD of the trigeminal nerve was effective in 7/9 SUNCT/SUNA patients (78%). Although longer follow-up is needed to establish the long term outcome of this procedure, these preliminary data support the role of MVD in the surgical management of SUNCT and SUNA.

No conflict of interest.

